# Protein function prediction through multi-view multi-label latent tensor reconstruction

**DOI:** 10.1186/s12859-024-05789-4

**Published:** 2024-05-02

**Authors:** Robert Ebo Armah-Sekum, Sandor Szedmak, Juho Rousu

**Affiliations:** Department of Computer Science, Aalto University, Konemiehentie 2, 02150 Espoo, Finland

**Keywords:** Protein function, Machine learning, CAFA, Gene ontology

## Abstract

**Background:**

In last two decades, the use of high-throughput sequencing technologies has accelerated the pace of discovery of proteins. However, due to the time and resource limitations of rigorous experimental functional characterization, the functions of a vast majority of them remain unknown. As a result, computational methods offering accurate, fast and large-scale assignment of functions to new and previously unannotated proteins are sought after. Leveraging the underlying associations between the multiplicity of features that describe proteins could reveal functional insights into the diverse roles of proteins and improve performance on the automatic function prediction task.

**Results:**

We present GO-LTR, a multi-view multi-label prediction model that relies on a high-order tensor approximation of model weights combined with non-linear activation functions. The model is capable of learning high-order relationships between multiple input views representing the proteins and predicting high-dimensional multi-label output consisting of protein functional categories. We demonstrate the competitiveness of our method on various performance measures. Experiments show that GO-LTR learns polynomial combinations between different protein features, resulting in improved performance. Additional investigations establish GO-LTR’s practical potential in assigning functions to proteins under diverse challenging scenarios: very low sequence similarity to previously observed sequences, rarely observed and highly specific terms in the gene ontology.

**Implementation:**

The code and data used for training GO-LTR is available at https://github.com/aalto-ics-kepaco/GO-LTR-prediction.

## Introduction

As one of the essential biomolecules in living cells, proteins perform a wide range of important functions including aiding cell division, supporting metabolism and providing immune response [18, 22]. Thus, a proficient knowledge of their functions is of crucial biological relevance, especially in elucidating metabolic pathways, understanding disease mechanisms and developing potent drugs. However, of the hundreds of millions of proteins that have been discovered and sequenced using high-throughput technologies, only a small proportion (< 1%) have been functionally characterized [6]. The huge disparity is mainly due to the time and resource constraints of experimental characterization techniques. As a result, computational methods offering fast, large-scale and accurate assignment of functional annotations are highly sought to bridge this ever-widening gap [6, 10].

Proteins are described by several characteristics ranging from the primary sequence, secondary structure, tertiary structure, chemical properties, to the physical interactions they have with other proteins in the performance of their functions [18, 22]. Consequently, several methods utilizing different protein feature sets have been developed, either in a single view or a multi-view setup [17, 28, 44]. Several approaches exist for integrating multiple input views including early, intermediate and late fusion methods. Current function prediction methods have used separate modules to learn salient features from respective feature sets [16, 21, 42] and merged the per-feature representations using concatenation or ranked the terms predicted by each feature-component method.

To actively advance the course of developing computational techniques for protein function annotation, the Critical Assessment of Functional Annotation (CAFA) challenge was introduced a decade ago [28]. Through this initiative, computational models developed are systematically assessed based on their accuracy in assigning functional annotations to new and previously uncharacterized proteins, on benchmark datasets curated from wet lab experiments. Evaluation is done using robust and standardized metrics developed by the community [15, 17, 44]. In CAFA, the gene ontology (GO), the most comprehensive resource for protein function annotations [3], is used to represent the functional categories and to evaluate the machine learning models. The thousands of GO categories give rise to a multi-label prediction problem where several GO categories may be valid for a single protein.

In this study, we address the function prediction problem by modeling the joint interactions between different features using the latent tensor reconstruction approach [35, 41], which can be viewed as an extension of higher-order factorization machines [7]. Building on the factorized parameterization and expressivity of factorization machines [7, 29] in modeling complex interactions between variables, LTR leverages the linear form factorization of tensors [19] and a mini-batch data processing scheme, thereby scaling to large datasets while maintaining constant memory and linear time complexity in the size of the input features as well as in the order, size and rank of the tensor. In summary, the study makes the following contributions:

- We present GO-LTR, a multi-view multi-label prediction model for automatic function annotation, based on the latent tensor reconstruction approach.
- We show that GO-LTR improves performance on the function prediction task, as assessed by multiple evaluation metrics.
- We show that leveraging multiple protein modalities, including the recent foundation models based on large language models, results in enhanced performance.
- We present detailed studies on the prediction performance, in terms of similarity to observed sequences in the training set, depth and frequency of GO classes, as well as the prediction threshold of the models.

## Methods

### Learning task

In the protein function prediction task, we are given a dataset $\{\mathscr{D}=(\textbf{x}_i, \textbf{y}_i)\mid i=1,\ldots ,m\}$. For each data sample, there are $n_d$ feature vectors, called the views, $\textbf{x}_{i}^{\left( d\right) }\mid \textbf{x}_{i}^{\left( d\right) }\in \mathbbm {R}^{n_{x_{d}}},\quad d=1,\ldots ,n_d$ of potentially different dimensions $n_{x_{d}}$, representing different representations of a protein, for example, sequence embeddings, InterPro fingerprints and protein-protein interaction embeddings. Each data sample is associated with a multi-label target vector, $\textbf{y}_i \in \{0,1\}^{n_{y}}$ denoting the membership of the example $\textbf{x}_i$ to the different functional categories, which in this work are taken from the gene ontology (GO).

In the matrix representation, each sample $\textbf{x}_{i}^{\left( d\right) }$ is embedded into a row of the input matrix $\textbf{X}^{\left( d\right) }$ belonging to view *d* and $\textbf{y}_i$ is a row of the label matrix $\textbf{Y}$.

### Latent tensor reconstruction (LTR)

We used the latent tensor reconstruction (LTR) model [35, 41] in our experiments (Table 1). LTR, similarly to higher-order factorization machines [7], is based on a tensor-based approximation of a degree $n_{d}$ polynomial function:

1 $$\begin{aligned} \begin{aligned} f(\textbf{x}) =\sum _{j=1}^{n} w_j x_j + \sum _{j,k=1}^{n} w_{jk} x_j x_k + \cdots + \sum _{j_1,j_2,\ldots , j_{n_d}=1}^{n} w_{{j_1},\ldots ,j_{n_d}} x_{{j_1},\ldots ,}x_{j_{n_d}} \end{aligned} \end{aligned}$$

As the number of parameters in the model is exponential in the polynomial degree $n_d$, instead a factorized representation (Table 1c), where each regression coefficient $w_{j_1,\dots ,j_r}$ is approximated by a weighted sum of products of factor weights, is used:

2 $$\begin{aligned} \begin{aligned} w_{j_1,\dots ,j_r} = \sum _{t=1}^{n_t} \lambda _t p_{j_1,t}\cdots p_{j_{r},t} \end{aligned} \end{aligned}$$

This trick provides an exponential reduction in the number of parameters that need to be estimated with both statistical and computational benefits.

**Table 1:** Computations in LTR model

Process	Computations
(a) Multiview data	Given: a sample $\mathscr{S} =((\textbf{x}_i^{(1)},\dots , \textbf{x}_i^{(n_d)}), \textbf{y}_i) \mid i\in [m]$, $\qquad \textbf{x}^{(d)}_i \in \mathbb {R}^{n_{x_d}},\ d\in [n_d],\quad \textbf{y} _i\in \mathbb {R}^{n_{y}}$ Output: Parameter tensor $\textbf{T}$
(b) Polynomial regression	$$\begin{array}{@{}ll@{}}&\displaystyle \min \limits _{\textbf{T}} \sum \limits _{i} \Vert y_i - \langle \textbf{T}, \otimes _{d=1}^{n_d} \textbf{x}_i^{(d)}\rangle \Vert ^2, \quad \text {scalar-valued case} \end{array}$$
(c) Tensor factorization, first level	$$\begin{array}{@{}ll@{}} \textbf{T} = \sum \limits _{t=1}^{n_t} \lambda _t \otimes _{d=1}^{n_d} \textbf{p}_{t}^{(d)} \\ \displaystyle \pi \left( \textbf{x}\right) = \sum \limits _{t=1}^{n_{t}} \lambda _{t} \langle \otimes _{d=1}^{n_{d}}\textbf{p}_{t}^{\left( d\right) }, \otimes _{d=1}^{n_{d}} \textbf{x}^{(d)} \rangle = \sum \limits _{t=1}^{n_{t}} \lambda _{t} \prod \limits _{d=1}^{n_{d}} \langle \textbf{p}_{t}^{\left( d\right) }, \textbf{x}^{(d)} \rangle \\ \qquad = \textbf{1}_{n_t}^{T}\textbf{D}_{\lambda } \circ _{d=1}^{n_d} \textbf{P}^{(d)}\textbf{x}^{(d)}, \ \textbf{D}_{\lambda } = {\textbf {diag}}\left( \lambda _{1}, \ldots , \lambda _{t}\right) \end{array}$$
(d) Tensor factorization, second level	$$\begin{array}{@{}l@{}l@{}} \displaystyle \textbf{P}^{(d)} = \textbf{V}^{(d)}\textbf{U}^{(d)T}\textbf{D}^{(d)}_{\lambda _{U}}, \quad \textbf{P}^{(d)} \in \mathbb {R}^{n_{t}\times n_{x_{d}}}\\ ||\textbf{V}^{(d)}_i||_2=1,\ i=[n_t],\ ||\textbf{U}^{(d)}_j||_2=1,\ j\in [n_{x_d}], \\ \qquad \textbf{D}_{\lambda _U} \text {diagonal} \\ \displaystyle \pi \left( \textbf{x}\right) = \textbf{1}_{n_t}^{T}\textbf{D}_{\lambda } \circ _{d=1}^{n_{d}} \left( \textbf{V}^{(d)} \textbf{U}^{(d)T}\textbf{D}^{(d)}_{\lambda _{U}}\textbf{x}\right) \end{array}$$
(e) Vector output for multi-labels	$$\begin{array}{ll} \displaystyle \varvec{\pi }\left( \textbf{x}\right) = \textbf{Q}^{T}\textbf{D}_{\lambda } \circ _{d=1}^{n_{d}} \left( \textbf{V}^{(d)} \textbf{U}^{(d)T}\textbf{D}^{(d)}_{\lambda _{U}}\textbf{x}\right) , \textbf{Q} \in \mathbb {R}^{n_{y}\times n_{t}} \end{array}$$
(f) Including activation functions, e.g., ReLU	$$\begin{array}{ll} \displaystyle \varvec{\pi }\left( \textbf{x}\right) = \textbf{Q}^{T}\textbf{D}_{\lambda } \circ _{d=1}^{n_{d}} \mathscr {B}\left( \textbf{V}^{(d)} \mathscr {A}\left( \textbf{U}^{(d)T}\textbf{D}^{(d)}_{\lambda _{U}}\textbf{x}\right) \right) \end{array}$$
(g) Inference	Given: data: $\textbf{x}$, parameters: $\textbf{Q}$, $\textbf{D}_{\lambda }$, ($\textbf{V}^{(d)}$, $\textbf{U}^{(d)}$, $\textbf{D}^{(d)}_{\lambda _U}$), $d=[n_d]$, Output: $\hat{\textbf{y}} = \varvec{\pi }(\textbf{x})$
(h) Optimization objective	$$\begin{array}{ll}\displaystyle \min \limits _{\varvec{\textbf{Q}, \lambda }, \textbf{V}^{(d)}, \textbf{U}^{(d)},\varvec{\lambda }_{U}^{(d)},d\in [n_d]} \quad \frac{1}{2mn_{y}}\Vert \textbf{Y} -\hat{\textbf{Y}}\Vert _{F}^{2} + \frac{C_{\lambda }}{2n_{t}}\Vert \varvec{\lambda }\Vert _{2}^{2} \\ \displaystyle \text {s.t. } ||\textbf{Q}_i||_2=1,\ i \in [n_t],\ ||\textbf{V}^{(d)}_i||_2=1,\ i\in [n_t],\\ \qquad ||\textbf{U}^{(d)}_j||_2=1,\ j \in [n_{x_d}],\ \textbf{D}_{\lambda _U}\ \text {diagonal} \\ \end{array}$$

[*m*] denotes the set $\{1,\ldots , m\}$, *m* refers to the number of data examples, $\langle \cdot \rangle$ denotes the inner product, and $\Vert \cdot \Vert$ represents the norm operator. $\otimes$ denotes the tensor product of vectors and $\circ$ connotes the pointwise multiplication of tensors of the same dimension. We use ${\textbf {y}}$ to denote a vector and ${\textbf {Y}}$ to represent a matrix

**Fig. 1:**
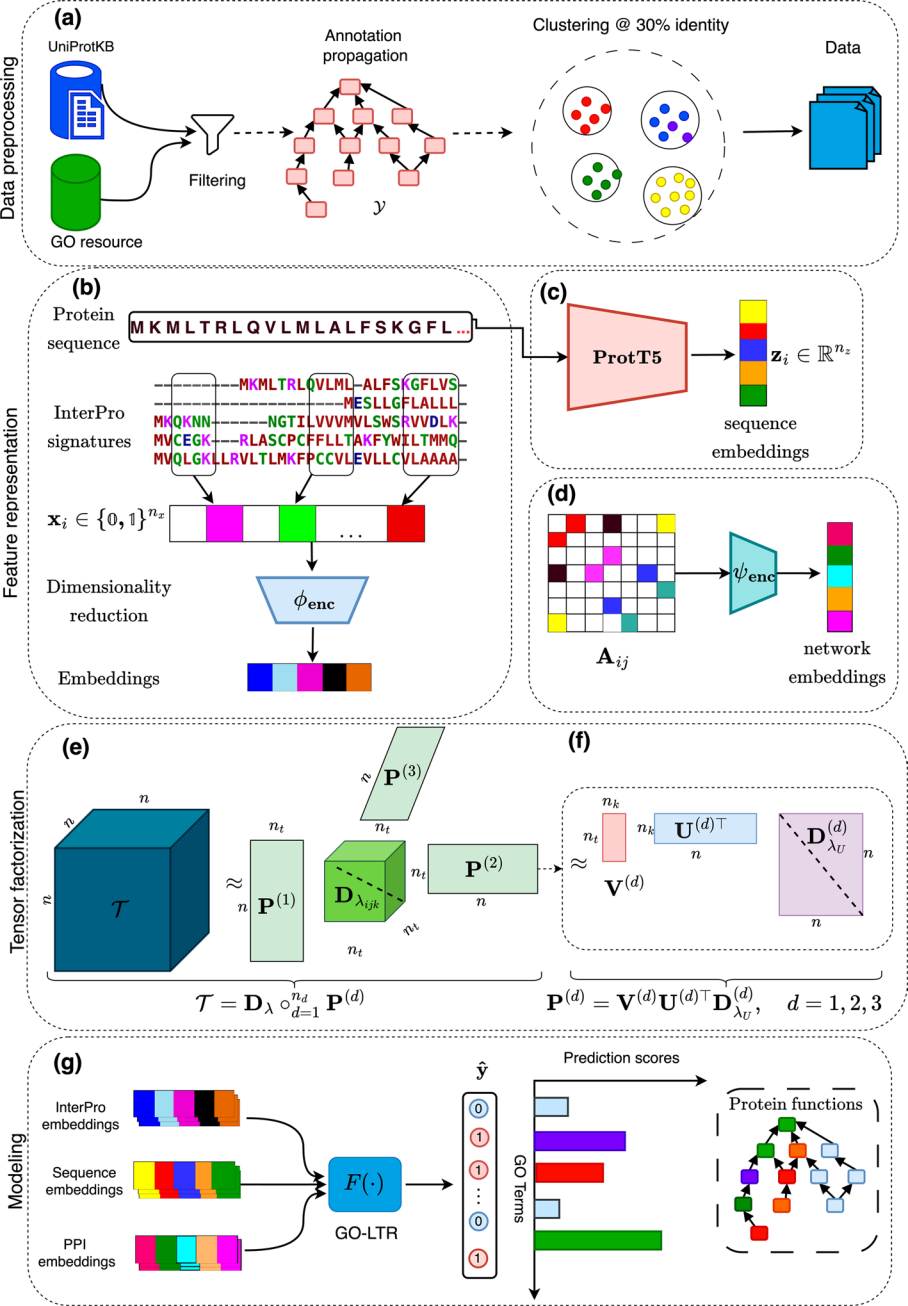
Project workflow: **a** Manually reviewed and annotated sequences are filtered based on term frequency after true-path propagation to ancestor terms. Using MMseqs2, sequences are clustered at 30% percentage identity. **b** Dimensionality reduction is performed on the binary vector of domain and family fingerprints from InterProScan. **c** Sequence embeddings of size 1024 are generated using ProtT5 protein language model. **d** Dimensionality reduction is applied on the rows of the adjacency matrix formed from the PPI network obtained from StringDB. **e** Parameter tensor decomposition in GO-LTR with $D_{\lambda _{ijk}}$ as a diagonal tensor, **f** further singular value decomposition of the parameter matrix $\textbf{P}^{\left( d\right) }$ of each feature (view) *d*. **g** GO-LTR predicts the scores associated with each functional term in the multi-label target vector using multiple features as input. The predicted score for each term is then propagated via the true-path rule to ensure prediction consistency such that each node retains the maximum score during the propagation process

In LTR, the parameter tensor $\textbf{T} = \sum \limits _{t=1}^{n_t} \lambda _t \otimes _{d=1}^{n_d} \textbf{p}_{t}^{(d)}$ collecting all the regression coefficients $w_{j_1,\dots ,r_r}$ is represented in factorized form as weighted sum of rank-one tensors (Fig. 1e). The factor matrices $\textbf{P}$ containing the factor weights of individual variables representation are further factorized through a singular value decomposition (Table 1d, Fig. 1f). This reparameterization has the effect of decoupling the factors representing individual variables and further decreasing the number of parameters to estimate.

LTR is capable of handling several, potentially heterogeneous data sources describing the same phenomenon, in a multi-view learning framework (Table 1a). For example, in the 3-view case studied in the experiments (“Experimental results on Dataset-1” section), cross-view interactions are modeled by the tensor product between the feature vectors of the views.

In the architecture, we introduce further non-linearity to enhance the representation power of the model by the use of rectified linear unit (ReLU) activation functions, $\mathscr {A}$ and $\mathscr {B}$ (Table 1f), applied on the linear layers. Notably, the use of activation functions generalizes the LTR model beyond polynomial functions, such as represented in Eq. 1.

To address the multi-label output problem, the vector $\textbf{1}_{n_{t}}$ of (Table 1c) is replaced by the learned matrix $\textbf{Q}$ in (Table 1e), which projects the vector-valued predictions into the output space. Finally, the optimization problem solves a regularised mean squared error between the ground truth $\textbf{Y}$ and prediction $\hat{\textbf{Y}}$ (Table 1h).

### Input data

In this study, we used the *“go-basic.obo”* ontology file [11] released on 1.1.2023, containing information about 46,739 terms. Additionally, we used the manually reviewed and annotated Swiss-Prot protein sequences in the Universal Protein Knowledgebase (UniprotKB) [5] which contains about half a million sequences (Fig. 1a). Following the CAFA rules for datasets curation [28], we present two time-separated datasets. Dataset-1 contains protein sequences annotated from the inception of UniprotKB up to 13.03.2023. Dataset-2 on the other hand is a collection of sequences annotated from the 14.03.2023 up to 24.01.2024. The data was filtered to remove duplicate sequences. Next, we selected sequences having at least one annotation supported by any of the following evidence codes: EXP, IDA, IPI, IMP, IGI, IEP, TAS, IC, HTP, HDA, HMP, HGI and HEP.

### Output data

The gene ontology (GO) provides a formal and comprehensive representation of the functions of gene products in living organisms using standardized and unified terminology [3]. Functions are described using three main subontologies representing three ancestral nodes: molecular function ontology (MFO), cellular component ontology (CCO) and biological process ontology (BPO).

Annotations are represented in a hierarchical format using a directed acyclic graph (DAG). Within this concept hierarchy, links between nodes are described using relations such as *is-a*, *part-of*, *negatively-regulates* and *capable-of*. Functional terms are related by the true-path propagation rule [4]—where a protein annotated to a deep-level node in the graph is automatically annotated to all its parent terms including the ancestral node(s). This implies that the set of functions associated with a particular protein forms a consistent sub-graph in the DAG.

In this work, we only considered *is-a* relationships and used the true path rule to propagate experimental annotations up to the root terms in the GO graph. The resulting sub-graph is then represented as a binary multi-label target vector. Functional terms having at least 30, 30 and 60 sequence examples were chosen as the final labels for MFO, CCO and BPO respectively. Table 2 provides a summary statistics of the final datasets for all three subontologies.

**Table 2:** Summary of datasets

Ontology	Terms	Size	
		Dataset-1	Dataset-2
MFO	776	29,650	849
CCO	615	37,025	785
BPO	3049	39,194	889

Terms do not include the ancestral nodes in the ontology

### Feature representation

We leveraged three data sources in our experiments—sequence embeddings, interpro fingerprints, and protein-protein interaction data.

Interpro fingerprints [2, 23, 24], encoding information about the motifs, active sites, conserved regions and protein families, were obtained from the Interpro service in the UniprotKB service. This is a binary fingerprint feature describing whether a particular subsequence/domain is present or absent in a sequence. We used an autoencoder composed of four layers interspersed with ReLU activation functions in both the encoder and decoder blocks to reduce the binary feature vector’s dimension from the highly sparse $\approx$14k to a dense representation vector of size 1000 (Fig. 1b).

We utilized sequence embeddings (1024 dimensions) generated using the ProtT5 [14] language model (Fig. 1c). Due to the computationally intensive nature of generating such dense representations, we downloaded the precomputed embeddings made available in the UniprotKB service for all sequences in our dataset. ProtT5 (Protein Text-to-Text Transfer Transformer) is a protein language model developed in [14] based on the transfomer architecture [37]. Analogous to natural language processing (NLP) models, ProtT5 considers each input amino acid (AA) sequence as a sentence and its constituent residues as tokens. It is trained in a self-supervised manner: learning to generate the sequence from the low-dimensional intermediate representations from an encoder (see Additional file 1: Fig. A11). The training data for ProtT5 spans over 300 billion amino acids from sequences sourced from large scale databases including big fantastic database (BFD) [12], UniRef50 [34] and UniRef100 [34]. Learning from only the sequence data, the latent representations given by ProtT5 encodes relevant information about proteins including domain, motifs and biophysical features compared to the expensive computation of multiple sequence alignments over large databases.

We also incorporated protein–protein interaction (PPI) network data from the StringDB database [36, 38]. The edges in this network denote the interactions between proteins in the performance of their functions, in a living cell. We create an N$\times$N adjacency matrix of the PPI graph using all N proteins in our dataset as nodes. Using an autoencoder, we performed dimensionality reduction on each row of the matrix to obtain a 1$\times$1000 dense representation vector (Fig. 1d).

### Baseline methods

In addition to commonly used baselines in the automatic function prediction tasks, we also considered machine learning methods that had an open-source implementation that we could train from scratch on our dataset.

#### BLAST—basic local alignment search tool

This method transfers annotations from sequences in the training set to the test set using sequence similarity computed from an optimal alignment between two sequences [1, 25, 32]. Spurious sequence alignments are filtered using an e-value of 0.001. We consider two variants below.

(i) *BLAST-full*—we transfer all annotations of the training sequence with the highest scoring alignment to a test sample via BLAST, as the multi-label prediction for the test sample in focus.
(ii) *BLAST-partial*—The prediction score for the *j*th microlabel of a particular test sample $\textbf{x}_{i}$ is calculated as the maximum sequence identity score of the test sample to all training sequences annotated with the term *j* [10].

#### Naive

Here, the relative frequency of a term in the training set is used as the prediction probability for the term in all protein sequences in the test set [10, 44].

#### DeepGOCNN

This utilizes a 1D convolutional neural network (CNN) to learn important features from a protein sequence. The input to the network is a one-hot representation of the amino acids in the protein’s primary sequence. It applies a linear projection layer on a series of 1D convolution operation using various filter sizes to capture relevant sub-sequences that are closely related to the function of the protein [20, 21].

#### DeepGOMLP

This method uses a multi-layer perceptron (MLP) network to annotate proteins. It consists of two perceptron blocks each consisting of a linear function, onto which a ReLU activation, batch normalization and dropout operations are applied in successive order. The output of the first block is connected to the output of the second block to maintain the flow of gradients during training. A sigmoid activation is finally applied to the representations learned by the feed-forward layers to produce a classification output. DeepGOMLP uses the full binary and highly sparse vector (>14k dimensions) of InterPro fingerprints as input to the network [20].

#### NetGO3.0

NetGO3.0 [40] is an upgraded version of state-of-the-art NetGO/NetGO2.0 [43] and GOLabeler [42] models developed in previous CAFA competitions. It consists of seven component methods: Naive, BLAST-KNN, Net-KNN, LR-3mer, LR-InterPro, LR-Text and LR-ESM. Naive assigns a term to a protein based on the empirical probability of the term in the training set. BLAST-KNN annotates a protein with a functional term based on its top-K BLAST hits. Net-KNN assigns functional terms to a protein based on the protein’s top-K interacting proteins from its PPI network data. Different logistic regression (LR) classifiers are trained for each label in the multi-label target vector using the frequency of amino acid trigrams in the protein’s amino acid sequence (LR-3mer), InterPro fingerprints (LR-InterPro) and text curated from research and protein databases (LR-text) respectively. LR-ESM trains a LR for each functional term using sequence embeddings generated by ESM1-b [30] protein language model. The predicted scores for each microlabel in each component method are ranked and the top-k ranked terms over all component models are chosen as the final prediction.

**Table 3:** Mathematical definitions of evaluation metrics: Below, $\tau$ is the prediction threshold, $Y_{i}$ is the groundtruth multilabel and $\hat{Y}_{i}$ is the predicted multilabel at threshold $\tau$, i.e. $\hat{Y}_i(\tau ) = \mathbbm {1}(\hat{Y}_i \ge \tau )$

Metric	Definition
Indicator function	$$\mathbbm {1}_{\mathscr {X}}(x) = {\left\{ \begin{array}{ll} 1 &{} \text {if } x \in \mathscr {X} \\ 0 &{} \text {if } x \notin \mathscr {X}\end{array}\right. }$$
Information content	$I( v ) = -\log _{2} P( v \mid \mathscr {P} a (v))$, $\mathscr {P}a(v)$ refers to the parent(s) of term *v* in the ontology.
Maximum $F_{1}$-score ($F_{max}$)	$$\displaystyle \begin{array}{@{}ll@{}} pr(\tau ) &{}= \frac{1}{m(\tau )} \sum \limits _{i=1}^{m(\tau )} \frac{\sum _{ v }\mathbbm {1}( v \in \{\hat{Y}_i(\tau ) \cap Y_i\})}{\sum _{ v }\mathbbm {1}( v \in \{\hat{Y}_i(\tau )\})}, \quad \text {precision} \\ rc(\tau ) &{}= \frac{1}{n_{y}} \sum \limits _{i=1}^{n_{y}(\tau )} \frac{\sum _{ v }\mathbbm {1}( v \in \{\hat{Y}_i(\tau ) \cap Y_i\})}{\sum _{ v }\mathbbm {1}( v \in \{Y_i\})}, \quad \text {recall} \\ F_{max} &{}= \max \limits _{\tau } \Biggl \{ 2\times \frac{pr(\tau )\times rc(\tau )}{pr(\tau )+rc(\tau )} \Biggr \} \end{array}$$
Weighted $F_{max}$ ($WF_{max}$)	$$\begin{array}{ll} \displaystyle wpr(\tau ) &{}= \frac{1}{m(\tau )} \sum \limits _{i=1}^{m(\tau )} \frac{\sum _{ v } I( v )\cdot \mathbbm {1}( v \in \{\hat{Y}_i(\tau ) \cap Y_i\})}{\sum _{ v }I( v ) \cdot \mathbbm {1}( v \in \{\hat{Y}_i(\tau )\})}, \text {weighted precision} \\ wrc(\tau ) &{}= \frac{1}{n_{y}(\tau )} \sum \limits _{i=1}^{n_{y}(\tau )} \frac{\sum _{ v } I( v )\cdot \mathbbm {1}( v \in \{\hat{Y}_i(\tau ) \cap Y_i\} )}{\sum _{ v } I( v )\cdot \mathbbm {1}( v \in \{Y_i\})}, \text { weighted recall} \\ WF_{max} &{}= \max \limits _{\tau } \Biggl \{ 2\times \frac{wpr(\tau )\times wrc(\tau )}{wpr(\tau )+wrc(\tau )} \Biggr \} \end{array}$$
Minimum semantic distance ($S_{min}$)	$$\begin{array}{ll} \displaystyle ru(\tau ) = \frac{1}{n_{y}}\sum \limits _{i=1}^{n_{y}} \sum \limits _{ v \in \{Y_i\}\backslash \{\hat{Y}_i(\tau )\}} I( v ), \quad \text {remaining uncertainty} \\ mi(\tau ) = \frac{1}{n_{y}}\sum \limits _{i=1}^{n_{y}} \sum \limits _{ v \in \{\hat{Y}_i\}\backslash \{Y_i(\tau )\}} I( v ), \quad \text {missing information} \\ S_{\min }(\tau ) = \min \limits _{\tau } \biggl \{ \sqrt{\bigl (ru^{2}(\tau ) + mi^{2}(\tau )\bigr )} \biggr \} \end{array}$$
Area under precision recall curve	$\text {AUPRC} = \sum \limits _{i=1}^{n_{\tau }} pr(\tau _{i}) \cdot (rc(\tau _{i}) - rc(\tau _{i-1})), \quad n_{\tau }\quad \text {is the number of thresholds}$
Area under receiver operating characteristics curve	$\text {AUROC} = \sum \limits _{i=1}^{n_{\tau }} TPR(\tau _{i}) \cdot (FPR(\tau _{i}) - FPR(\tau _{i-1})),$ TPR and FPR denote True and False positive rates respectively

$m(\tau )$ denotes the number of proteins in the test set for which one of the predicted scores is at least $\tau$, $n_y$ is the size of the test set and $n_Y$ denotes the dimension of the multi-label target vector

### Evaluation metrics

We used standard CAFA evaluation metrics in our experiments [10, 15, 28, 44]. Mathematical definitions for all evaluation metrics are summarised in Table 3. We assessed model performance using maximum $F_1$-score ($F_{max}$). From the precision-recall curve at varying decision thresholds, the $F_{max}$ is calculated as the harmonic mean of the precision and recall point that gives the highest $F_1$-score. This score reflects the pronounced class-imbalance in the dataset. We also report the ability of models to correctly predict the positive terms in the test set using the area under the precision-recall (PR) curve (AUPRC) metric. The ability of models to discriminate between the positive and negative classes at varying prediction thresholds, is also assessed using the area under the receiver operating characteristics (AUROC) curve [13].

Additionally, we compared model performance based on weighted $F_{max}$, ($WF_{max}$) and minimum semantic distance ($S_{min}$). These metrics weight predicted terms by their information content, taking into account the hierarchical nature of the ontology. Large importance is placed on highly specific terms while little importance is given to less specific labels [10, 26, 27]. In the computation of the conditional information content for each term, we followed the true-path annotation rule and calculated each term’s empirical distribution as the relative frequency of the term in the dataset, given that its parent terms are also annotated.

## Results

Here, we present the outcomes from the experimental validation of our model. We contrast our model’s performance with commonly adopted baselines in CAFA competitions—first, with BLAST, which leverages sequence similarity [1, 25, 32], and second, with a frequency-based approach, termed Naive [10]. Additionally, we compare our model’s predictive accuracy with state-of-the-art methods in protein function prediction—DeepGOCNN, DeepGOMLP [20, 21] and NetGO3.0 [40].

We present the experimental results on Dataset-1 and Dataset-2 in “Experimental results on Dataset-1” and “Experimental results on Dataset-2” sections respectively. Considering that the time of release of NetGO3.0 overlaps with the period for the curation of Dataset-1, we do not include comparison to NetGO3.0 in the results for Dataset-1. This is due to the fact that it is only available as a webserver, hence we are unable to guarantee that the sequences in Dataset-1 set do not overlap with those used in training the NetGO3.0 model. On Dataset-2, however, we show comparison to the NetGO3.0 model.

**Table 4:** Performance evaluation based on area under precision recall curve, (AUPRC, $\uparrow$ higher the better) and maximum $F_1$-score, ($F_{max}, \uparrow$ higher the better)

Model	${F_{max}} (\uparrow )$			AUPRC $(\uparrow )$		
	MFO	CCO	BPO	MFO	CCO	BPO
Naive	$0.401_{\text { {(0.007)}}}$	$0.619_{\text { {(0.007)}}}$	$0.347_{\text { {(0.004)}}}$	$0.177_{\text { {(0.005)}}}$	$0.429_{\text { {(0.007)}}}$	$0.231_{\text { {(0.006)}}}$
BLAST-partial	$0.443_{\text { {(0.013)}}}$	$0.415_{\text { {(0.007)}}}$	$0.282_{\text { {(0.008)}}}$	$0.284_{\text { {(0.009)}}}$	$0.258_{\text { {(0.007)}}}$	$0.142_{\text { {(0.005)}}}$
BLAST-full	$0.545_{\text { {(0.013)}}}$	$0.578_{\text { {(0.025)}}}$	$0.357_{\text { {(0.012)}}}$	–	–	–
DeepGOCNN	$0.501_{\text { {(0.011)}}}$	$0.655_{\text { {(0.005)}}}$	$0.376_{\text { {(0.005)}}}$	$0.284_{\text { {(0.007)}}}$	$0.207_{\text { {(0.011)}}}$	$0.268_{\text { {(0.003)}}}$
DeepGOMLP	$0.673_{\text { {(0.008)}}}$	$0.657_{\text { {(0.006)}}}$	$0.454_{\text { {(0.005)}}}$	$0.498_{\text { {(0.014)}}}$	$0.475_{\text { {(0.013)}}}$	$0.397_{\text { {(0.007)}}}$
GO-LTR	${\textbf {0.682}}_{\text { {(0.007)}}}$	${\textbf {0.722}}_{\text { {(0.006)}}}$	${\textbf {0.486}}_{\text { {(0.006)}}}$	${\textbf {0.716}}_{\text { {(0.009)}}}$	${\textbf {0.787}}_{\text { {(0.006)}}}$	${\textbf {0.481}}_{\text { {(0.007)}}}$

Metrics are reported as mean ± Standard Deviations (SDs) in the form $\text {mean}_{\text { {(SD)}}}$ over 10 Cross Validation (CV) folds in all 3 ontologies. AUPRC is not reported for BLAST-full since it outputs only binary-valued predictions. A perfect prediction has $F_{max}=1$ and AUPRC = 1. Best performing models are indicated in bold font

**Table 5:** Performance evaluation based on weighted maximum $F_1$-score, ($WF_{max}, \uparrow$ higher the better) and minimum semantic distance ($S_{min}, \downarrow$ lower the better)

Model	${WF_{max}}(\uparrow )$			${S_{min}}(\downarrow )$		
	MFO	CCO	BPO	MFO	CCO	BPO
Naive	$0.198_{\text { {(0.016)}}}$	$0.320_{\text { {(0.036)}}}$	$0.258_{\text { {(0.009)}}}$	$11.9_{\text { {(0.310)}}}$	$10.9_{\text { {(0.321)}}}$	$40.0_{\text { {(1.76)}}}$
BLAST-partial	$0.383_{\text { {(0.017)}}}$	$0.292_{\text { {(0.010)}}}$	$0.230_{\text { {(0.006)}}}$	$71.4_{\text { {(11.3)}}}$	$84.3_{\text { {(8.78)}}}$	$408.0_{\text { {(85.6)}}}$
BLAST-full	$0.443_{\text { {(0.013)}}}$	$0.380_{\text { {(0.022)}}}$	$0.263_{\text { {(0.010)}}}$	$11.5_{\text { {(1.09)}}}$	$12.3_{\text { {(1.64)}}}$	$46.1_{\text { {(1.64)}}}$
DeepGOCNN	$0.344_{\text { {(0.015)}}}$	$0.434_{\text { {(0.006)}}}$	$0.274_{\text { {(0.005)}}}$	$11.1_{\text { {(0.204)}}}$	$10.1_{\text { {(0.210)}}}$	$40.6_{\text { {(1.73)}}}$
DeepGOMLP	$0.586_{\text { {(0.010)}}}$	$0.464_{\text { {(0.006)}}}$	$0.367_{\text { {(0.006)}}}$	$8.17_{\text { {(0.311)}}}$	$9.74_{\text { {(0.218)}}}$	$37.7_{\text { {(1.15)}}}$
GO-LTR	${\textbf {0.591}}_{\text { {(0.008)}}}$	${\textbf {0.573}}_{\text { {(0.009)}}}$	${\textbf {0.392}}_{\text { {(0.004)}}}$	${\textbf {7.92}}_{\text { {(0.263)}}}$	${\textbf {8.16}}_{\text { {(0.214)}}}$	${\textbf {34.5}}_{\text { {(1.37)}}}$

Metrics are reported in the form $\text {mean}_{\text { {(SD)}}}$ over 10 CV folds in all 3 ontologies. Prediction scores for terms are weighted by their conditional information content in the dataset. Thus, higher weights are given to more informative, deep and specific terms. Best performing models are highlighted in bold font

### Experimental results on Dataset-1

#### Cross-validation setup for Dataset-1

In order to reduce the risk of exaggerating generalization performance [39], we performed a homology separation between the train and test sets. We clustered the sequences in Dataset-1 at a 30% sequence identity cut-off using mmseqs2 [33]. Proteins within a cluster have at least 30% sequence similarity and 60% coverage with the cluster representative (i.e centroid). This enforces a between-cluster similarity of < 30%. Clusters are iteratively refined to improve the within-cluster homology as measured by the sequence similarity. We then randomly selected 90% of the clusters for training and 10% for testing in a 10-fold cross-validation setting. All proteins in a cluster are wholly included in the train or test set. We trained models separately for each ontology. Models are optimized using 10-fold cross-validation. 10% of the sequences in the training set are used as a validation set. After the parameter optimization process for each fold, we then retrained the models on the full training set and evaluated the performance on the held-out test set.

#### Performance comparison of GO-LTR and competing methods

As shown in Table 4, we evaluated the predictive accuracy of our model using maximum $F_{1}$-score ($F_{max}$), one of the metrics used in CAFA evaluations. Consistent with previous studies, it is evident that the machine learning (ML) methods (DeepGOCNN, DeepGOMLP and GO-LTR) outperform the common baselines used by the function prediction community [17, 28, 44]. GO-LTR recorded the best performance compared to all competing methods in all 3 categories. In MFO, GO-LTR slightly outperformed DeepGOMLP, the second best method, with a marginal 1.3% difference in $F_{max}$. In predicting the cellular locations in the CCO category, GO-LTR recorded a significant performance improvement (0.718) over second-placed DeepGOMLP (0.657) model. In BPO, however, all models recorded $F_{max}$ below 0.5, the worst performance compared to MFO and CCO function categories. This can be attributed to the dense and highly unbalanced nature of the BPO subontology. Also, the BPO graph mainly comprises broad and highly-unspecified terms. Additionally, homology-based BLAST methods were better at transferring functional terms than frequency-based Naive in MFO, but not in CCO nor BPO. Due to the highly-skewed nature of the GO dataset, we also assessed our model’s performance using the area under precision recall curve (AUPRC). From Table 4, it is evident that GO-LTR’s ability to predict the positive classes in the test set far exceeds that of all other competing methods. Although only modest performance differences are seen between GO-LTR and the ML baselines under the $F_{max}$ metric, GO-LTR’s strong classification performance is seen more clearly under the AUPRC metric. This indicates that GO-LTR is highly robust with respect to the choice of the prediction threshold between positive and negative classes. The Precision-Recall and ROC curves for all models are shown in Additional file 1: Figs. A7 and A8.

#### Information-theoretic assessment of model performance

In Table 5, we compared the predictive accuracy of models using information theoretic measures, weighted $F_{max}$ and minimum semantic distance ($S_{min}$). As expected, it is seen that weighting predicted terms by their conditional information content resulted in a reduction in model performance from higher values in $F_{max}$ in Table 4 to moderately lower values in $WF_{max}$ in Table 5. For instance, GO-LTR’s performance dropped from an $F_{max}$ of 0.682 to a $WF_{max}$ of 0.593. Even after the inclusion of each term’s information content, GO-LTR still showed an advantage over all other methods in predicting highly specific terms in the ontology. Considering the $S_{min}$ metric, BLAST-partial showed the least promise in identifying labels of high informative value, manifesting even worse performances in the relatively easy cases of MFO and CCO.

**Table 6:** Ablation experiment: Effect of feature combinations on GO-LTR performance as measured by $F_{max}$, $\uparrow$ higher the better, in all 3 ontologies

Views	${F_{max}} (\uparrow )$			
	Feature combinations	MFO	CCO	BPO
1-View	$$\begin{array}{l}\text {InterPro}\\ \text {PPI} \\ \text {UniProt} \\ \end{array}$$	$$\begin{array}{c} 0.610_{\text { {(0.010)}}}\\ 0.491_{\text { {(0.009)}}} \\ 0.651_{\text { {(0.010)}}} \\ \end{array}$$	$$\begin{array}{c} 0.657_{\text { {(0.006)}}}\\ 0.661_{\text { {(0.007)}}} \\ 0.708_{\text { {(0.005)}}} \\ \end{array}$$	$$\begin{array}{c} 0.409_{\text { {(0.005)}}}\\ 0.389_{\text { {(0.006)}}} \\ 0.463_{\text { {(0.005)}}} \\ \end{array}$$
2-View	$$\begin{array}{l}\text {InterPro + PPI}\\ \text {InterPro + UniProt} \\ \text {PPI + UniProt}\\ \end{array}$$	$$\begin{array}{c} 0.644_{\text { {(0.009)}}}\\ {\textbf {0.682}}_{\text { {(0.008)}}} \\ 0.670_{\text { {(0.009)}}} \\ \end{array}$$	$$\begin{array}{c} 0.682_{\text { {(0.006)}}}\\ 0.710_{\text { {(0.006)}}} \\ {\textbf {0.722}}_{\text { {(0.006)}}} \\ \end{array}$$	$$\begin{array}{c} 0.445_{\text { {(0.006)}}}\\ 0.481_{\text { {(0.006)}}} \\ 0.484_{\text { {(0.006)}}} \\ \end{array}$$
3-View	$$\begin{array}{l}\text {InterPro + PPI + UniProt}\end{array}$$	$$\begin{array}{c}{} {\textbf {0.682}}_{\text { {(0.007)}}} \end{array}$$	$$\begin{array}{c}0.718_{\text { {(0.006)}}}\end{array}$$	$$\begin{array}{c}{} {\textbf {0.486}}_{\text { {(0.006)}}}\end{array}$$

Metric is reported as $\text {mean}_{\text { {(SD)}}}$ over 10 CV folds. Best performing feature combinations are highlighted in bold font

**Table 7:** Ablation experiment: Performance comparison of machine learning models using all 3 features as input

Model	${F_{max}} (\uparrow )$			AUPRC $(\uparrow )$		
	MFO	CCO	BPO	MFO	CCO	BPO
CNN-3-view	$0.561_{\text { {(0.010)}}}$	$0.671_{\text { {(0.006)}}}$	$0.415_{\text { {(0.007)}}}$	$0.314_{\text { {(0.010)}}}$	$0.194_{\text { {(0.007)}}}$	$0.281_{\text { {(0.006)}}}$
MLP-3-view	${\textbf {0.689}}_{\text { {(0.007)}}}$	${\textbf {0.726}}_{\text { {(0.006)}}}$	${\textbf {0.500}}_{\text { {(0.006)}}}$	$0.615_{\text { {(0.010)}}}$	$0.587_{\text { {(0.008)}}}$	${\textbf {0.487}}_{\text { {(0.009)}}}$
LTR-3-view	$0.681_{\text { {(0.007)}}}$	$0.718_{\text { {(0.006)}}}$	$0.486_{\text { {(0.006)}}}$	${\textbf {0.710}}_{\text { {(0.010)}}}$	${\textbf {0.779}}_{\text { {(0.006)}}}$	$0.481_{\text { {(0.007)}}}$

Evaluation metrics are reported as $\text {mean}_{\text { {(SD)}}}$ over 10 CV folds in all 3 ontologies. Best performing models are indicated in bold font

#### Contribution of different features to GO-LTR’s performance

We analysed the contribution of different features to GO-LTR’s predictive performance. We note that the combination of features in a multi-view learning paradigm could have complementary, redundant or contradictory effects. The results of this analyses are summarised in Table 6. The best performing GO-LTR model in MFO utilized a combination of InterPro fingerprints and sequence embeddings (UniProt). Although the third-order GO-LTR model exploiting all 3 features had the same predictive accuracy as its second-order (2-view) counterpart using InterPro and UniProt, we chose the latter model owing to its parsimonious nature. Using the network data alone (PPI) resulted in the worst performance in the MFO branch of the ontology. In the CCO category, where the goal is predicting the cellular location of proteins, it is seen that PPI had a better predictive accuracy compared to InterPro. This improved performance compared to that in MFO corroborates the assertion that proteins working together tend to be situated in close proximity to one another. Combination of all 3 features, however, did not yield any substantial improvement over the best performing 2-view model in the CCO category. Notably, we see that the best performing model in BPO used a combination of all three features. Indeed, all features were important in predicting terms in the BPO graph owing to its inherent complexity and dense nature. In consonance with previous works [8, 17, 28], we see that the sequence embeddings (UniProt) had the most prominent predictive signal compared to the other 2 features in all 3 ontology categories. The results in Additional file 1: Table A4 and Figs. A2, A3 and A4 further highlights the contributions of each feature to the predictive accuracies of the 2-view and 3-view GO-LTR models.

#### Performance comparison using all 3 features in ML-based models

Next, we investigated the predictive accuracy of competing ML models by using all 3 features as input to the models. The results are presented in Table 7. In MLP-3-view, the concatenation of all 3 features was used as the new input to the original DeepGOMLP model. In respect of CNN-3-view, we concatenated the features learned by the top layers of the CNN architecture from the 1D protein sequence with the InterPro fingerprints and the PPI data. This new representation was then passed as input to the subsequent layers of the DeepGOCNN model. It is seen that the exploitation of different features enhanced the predictive accuracies of both DeepGO models. Specifically, we see substantial improvement of $\approx$20% and $\approx$5% in the AUPRC of DeepGOMLP and DeepGOCNN respectively. This implies that the underlying associations between the features learned by the models resulted in improved precision and recall compared to their 1-view equivalents reported in Table 4. As shown in Table 7, MLP-3-view had the best performance in all 3 ontologies, closely followed by its GO-LTR counterpart. Although GO-LTR learns the explicit polynomial interaction between features, it achieves a similar performance to MLP-3-view which leveraged a concatenation of all 3 feature sets as input. The accompanying plots for the PR and ROC curves are shown in Additional file 1: Figs. A9 and A10.

**Fig. 2:**
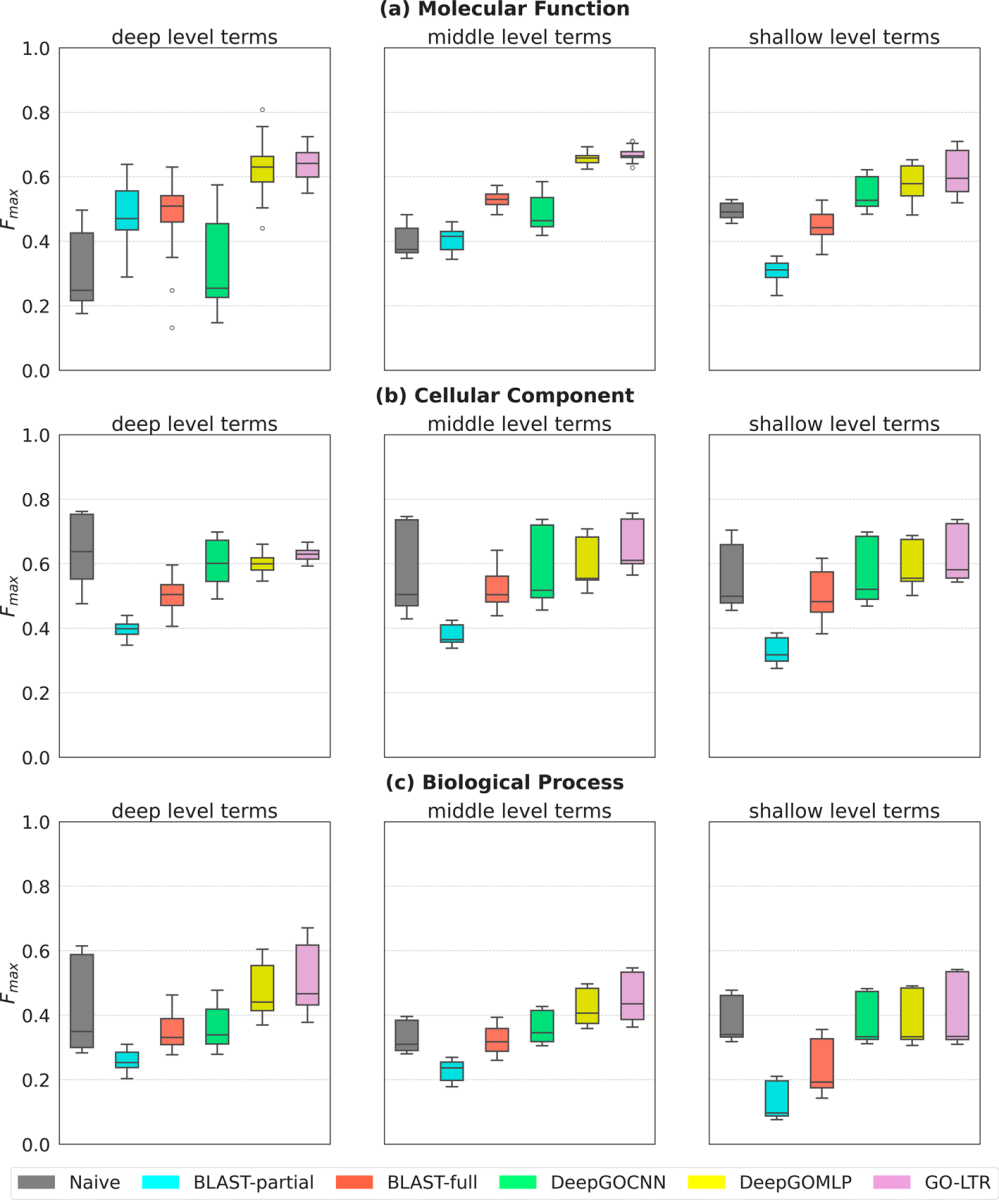
Performance evaluation based on depth of terms in the training sets of Dataset-1 for **a** molecular function ontology; **b** cellular component ontology; **c** biological process ontology. The horizontal line drawn in the box plots denote the median $F_{max}$ score while the lower and upper whiskers are the respective minimum and maximum values. The lower and upper hinges of the box reflect the 25th and 75th percentiles respectively. The points outside the minimum and maximum are the outliers. The greater the median the better the performance

**Fig. 3:**
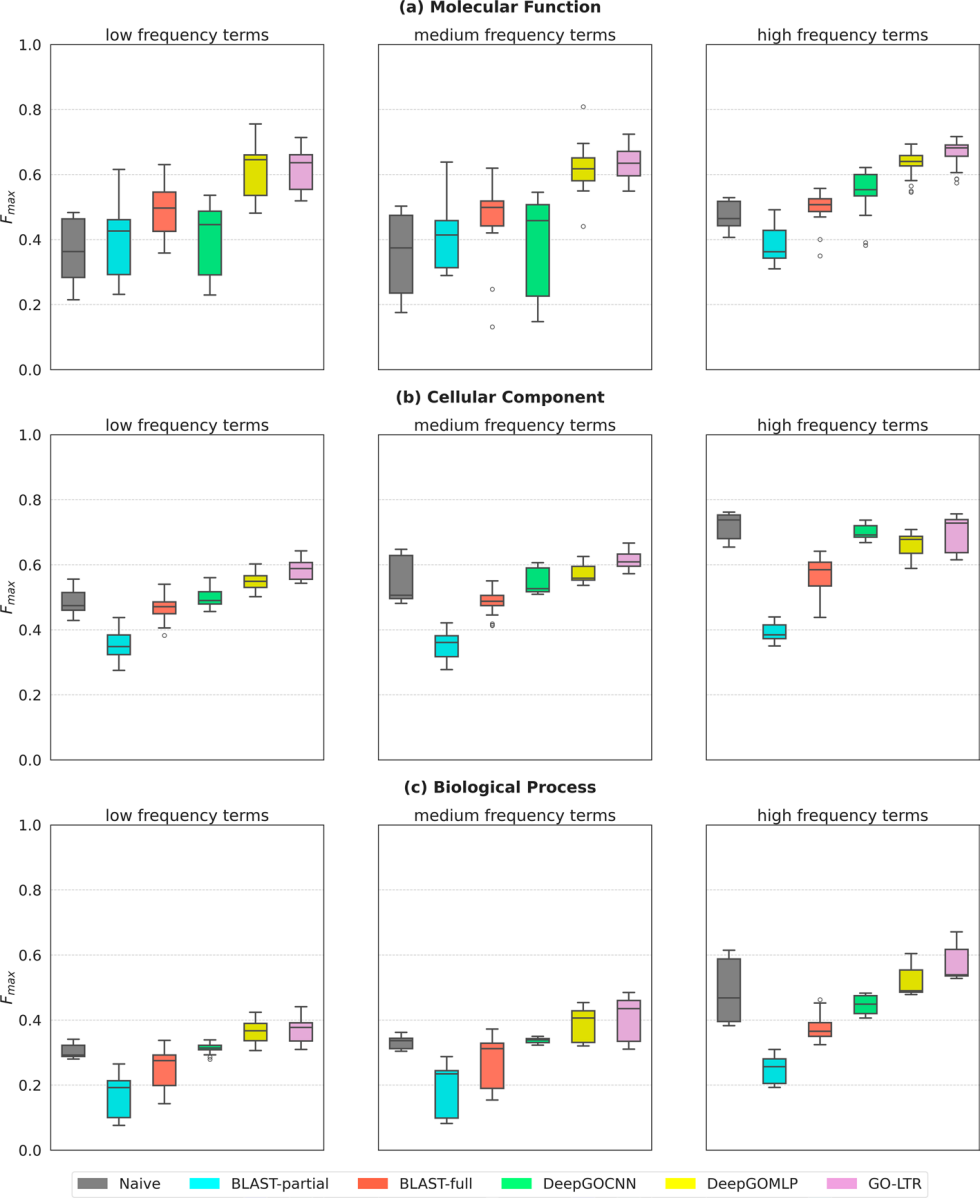
Performance comparison based on term frequencies in the training sets of Dataset-1 for **a** molecular function ontology; **b** cellular component ontology; **c** biological process ontology. The horizontal line drawn in the box plots denote the median $F_{max}$ score while the lower and upper whiskers are the respective minimum and maximum values. The lower and upper hinges of the box reflect the 25th and 75th percentiles respectively. The points outside the minimum and maximum are the outliers. The greater the median the better the performance

#### Performance evaluation on subset of terms: depth categorizations

Further, we studied the annotation accuracy of models on different subset of labels considering their depths in the ontology. In Fig. 2, we compared the performance of all models on different subset of terms differentiated by their depths in the ontology. Terms located on depths 8–11 were chosen as deep level terms, those on depths 3–7 were selected as middle level terms and labels above the third level were considered shallow level nodes. In MFO, GO-LTR outperformed all models across all 3 depth categories in the ontology (Fig 2a). In CCO, however, the Naive model had the best performance on deep level terms, closely followed by GO-LTR (Fig 2b). GO-LTR recorded the highest accuracy on the middle and shallow zones of the CCO ontology. Similarly, in BPO, Naive showed a competitive performance, even outperforming all models in predicting the shallow level nodes (Fig 2c). GO-LTR recorded the best accuracy in predicting nodes located in the deep and middle zones of the BPO category.

#### Performance evaluation on subset of terms: frequency categorizations

Furthermore, we evaluated the performance of all models in predicting subsets of terms grouped by their annotation frequency in the training set. Here, terms with <100 sequence examples were categorized as low frequency labels, those with frequencies in the range [100, 500) were labelled as medium frequency labels and terms having >500 examples were chosen as high frequency labels. In Fig. 3a, GO-LTR showed competitive performance across all 3 frequency groupings with small variations in the performance over the 10 cross validation folds. As shown in Fig. 3b, we see that frequency-based Naive model outperformed all models for subset of highly frequent terms in the CCO category. This means that the term frequency alone contains a high signal for predicting such terms. Hence, an appropriate combination of the predictions of machine learning and frequency-based methods could lead to performance improvement. GO-LTR exhibited the best performance on all frequency classes in the BPO ontology (Fig. 3c).

#### Performance stability analyses based on optimal prediction threshold

**Fig. 4:**
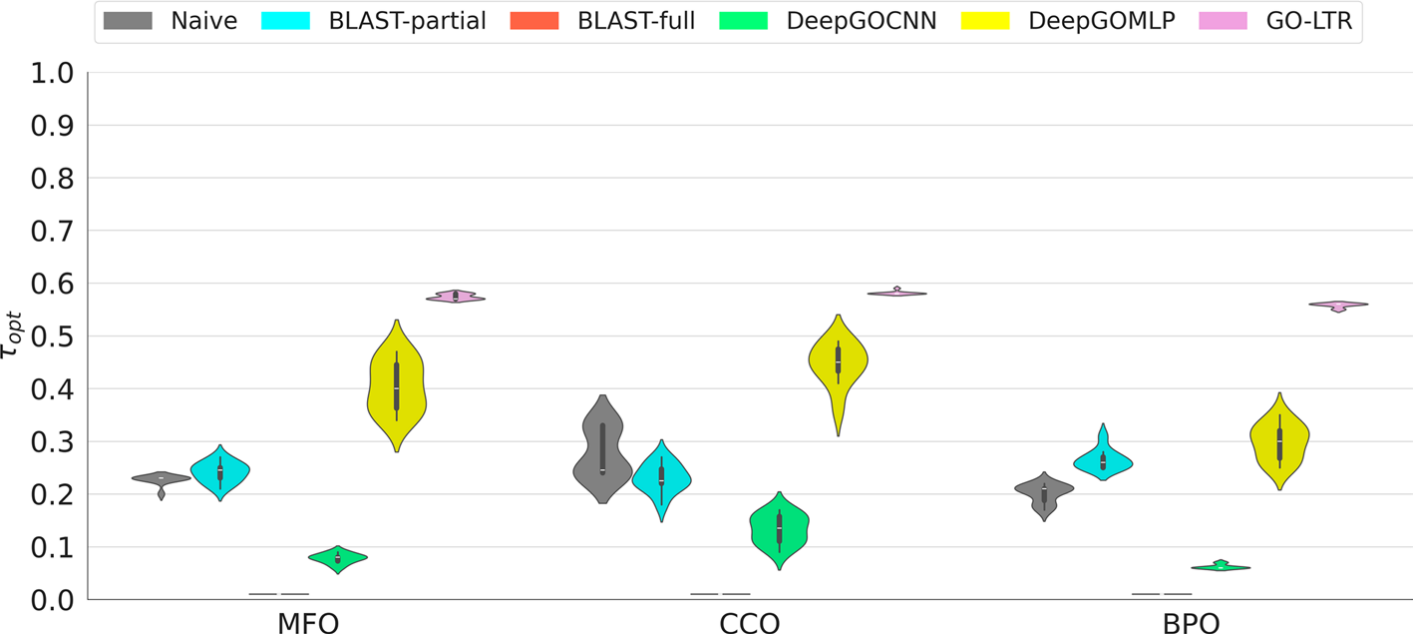
Performance stability evaluation using optimal prediction threshold in all 3 subontologies based on 10-fold cross validation on Dataset-1. Threshold for BLAST-full is not reported as it outputs only binary predictions. The shorter the height of the violin plot, the lesser the variability in the optimal decision threshold

Due to the extreme class-imbalance in the dataset, an adjustment to the decision threshold is necessary to reflect this bias and obtain optimal performance. As such, we assessed the stability of model predictions using the optimal prediction threshold ($\tau _{opt}$), the threshold yielding the maximum F_1_-score. This analysis gives an overview of a model’s robustness and sensitivity to small perturbations in the underlying data. As shown in Fig. 4, GO-LTR exhibited a highly stable performance across the 10 cross validation (CV) folds in all 3 ontologies. DeepGO methods on the other hand recorded relatively large fluctuations in the optimal prediction threshold, making them prone to distributional shifts. We hypothesize that the huge sizes of DeepGO models as depicted by the number of trainable model parameters (Additional file 1: Table A5) may account for their relatively high variances compared to small-sized GO-LTR.

### Experimental results on Dataset-2

Here, models are retrained on sequences in Dataset-1 and predictions are made for proteins in Dataset-2. Hence, Dataset-2 is used as an unseen dataset on which generalization performance is assessed. Models are trained separately for each ontology. Additionally, we submitted sequences in Dataset-2 to the NetGO3.0 webserver and recorded the results.

#### Performance evaluation on Dataset-2

**Fig. 5:**
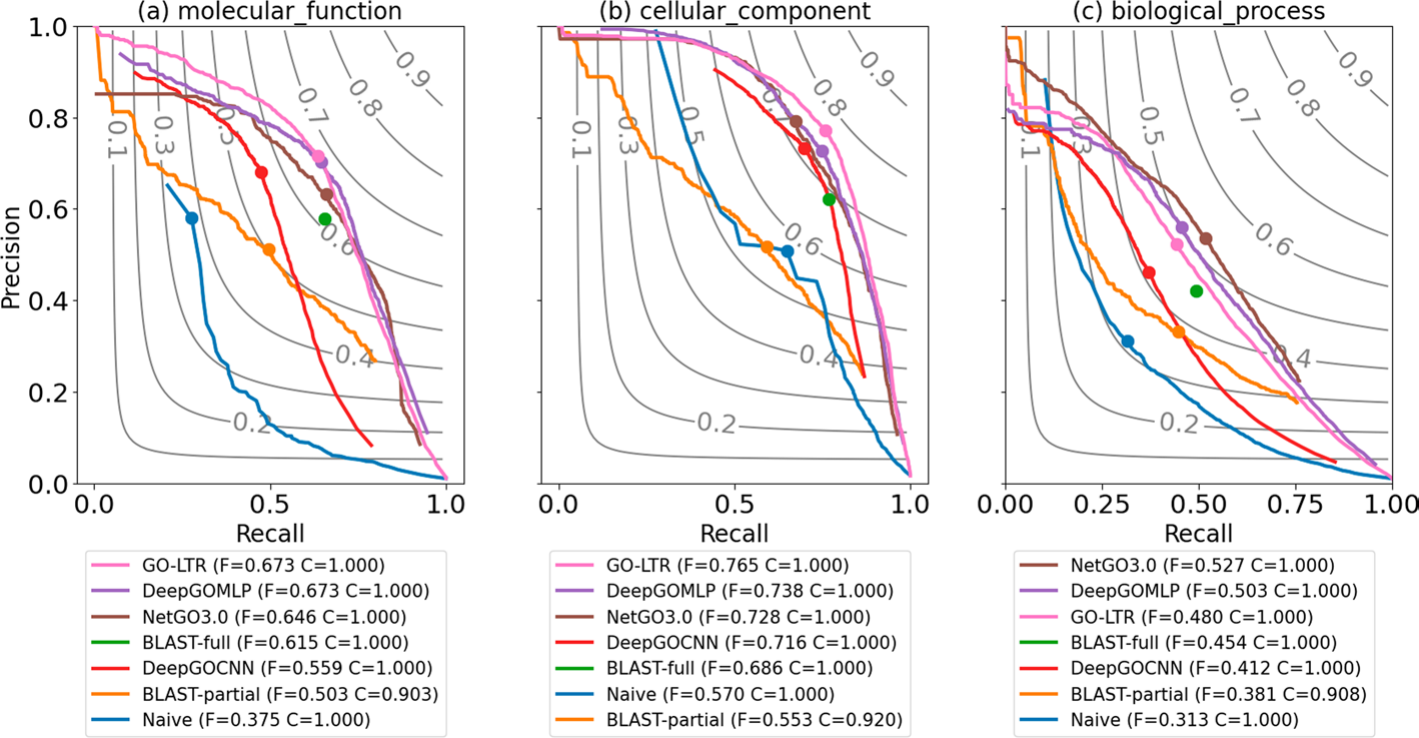
Performance comparison on Dataset-2 using $F_{max}(\uparrow )$ in **a** molecular function, **b** cellular component and **c** biological process ontology. The dot on the precision-recall curves indicate the precision-recall point at which the $F_{max}$ was achieved. The perfect model should have $F_{max} = 1$ at the top-right corner of the plot. In the legends, the $F_{max}$ (F) and the coverage (C) for each model is reported. Coverage refers to the number of proteins in the test set for which the model made non-zero predictions for at least one functional term

We compared the generalization performance of all models on an independent test set, Dataset-2. From the precision-recall curves in Fig. 5, GO-LTR achieves competitive annotation accuracy in the MFO category, performing on par with the DeepGOMLP model. In CCO, however, GO-LTR’s performance surpassed all other models. In BPO, NetGO3.0, which leverages multiple features ranging from research text, term frequency, sequence embeddings, PPI, InterPro and sequence-similarity-based annotation transfer, came in first place, outperforming both DeepGOMLP and GO-LTR. The strong performance exhibited by multi-modal NetGO3.0 and the results for 3-view GO-LTR in Additional file 1: Table A4 highlights the crucial importance of multi-view methods in improving annotation accuracy on the BPO category of the gene ontology. From the results of information theoretic performance evaluation presented in Additional file 1: Tables A1–A3, we see that GO-LTR exhibits a strong potential in predicting highly specific and rarely annotated terms in the various ontologies. In the ROC space, where a model’s ability to discriminate between positive and negative classes is assessed, GO-LTR, again, shows a highly competitive generalization performance (Additional file 1: Fig. A1).

#### Performance comparison based on maximum sequence identity

**Fig. 6:**
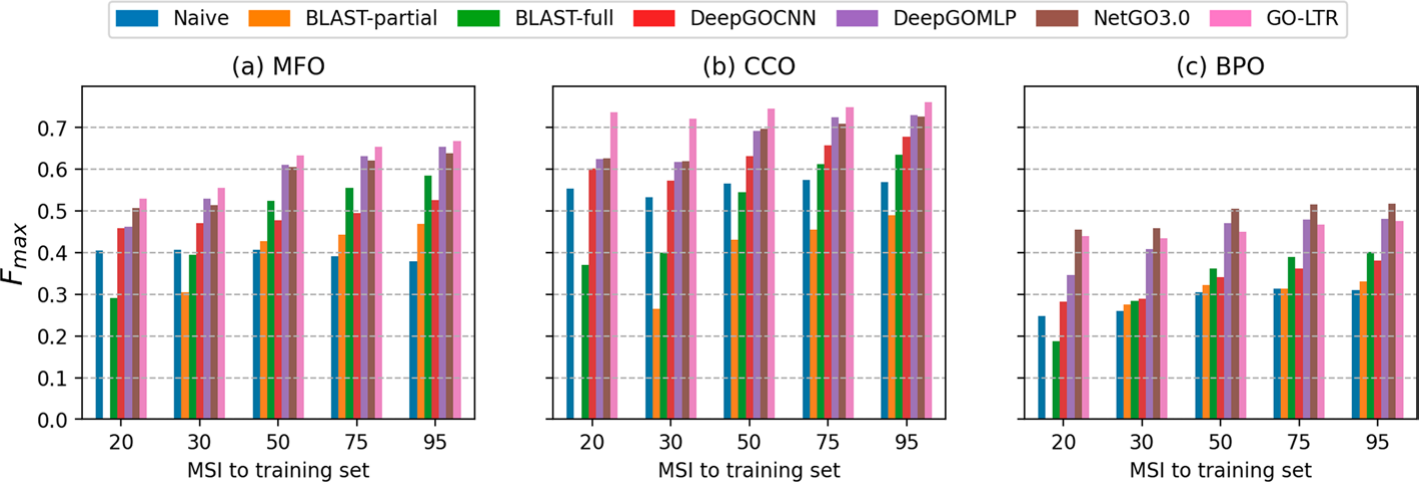
Performance comparison on Dataset-2 using $F_{max}(\uparrow )$ based on groupings of sequences in the test set by their maximum percentage sequence identity (MSI) to sequences in the training set, in **a** molecular function ontology, **b** cellular component ontology and **c** biological process ontology. The absence of BLAST-partial in the 20% MSI cutoff is due to the absence of relevant hits among training sequences detected at an e-value of 0.001

We investigated the practical utility of GO-LTR versus all competing methods in annotating novel sequences at varying degrees of homology to the sequences present in the training set. We partitioned the sequences in the test set into 5 groups ($\le 20\%$, $\le 30\%$, $\le 50\%$, $\le 75\%$ and $\le 95\%$) based on their maximum percentage sequence identity (MSI) to those in the training set. As illustrated in Fig. 6a, GO-LTR achieved the best generalization performance across all sequence similarity thresholds in the MFO category, including the highest performance on sequences with very low homology ($\le 20\%$ and $\le 30\%$) to known sequences in the training set. Similar results asserting the good performance of GO-LTR are shown in Fig. 6b for the CCO function category. In BPO, however, NetGO3.0 recorded the highest performance, followed closely by GO-LTR for very low sequence similarity cutoffs (Fig. 6c). DeepGOMLP edged past GO-LTR slightly for sequences with relatively high homology ($>50\%$ similarity) to the training set. As anticipated, similarity-based BLAST recorded worse performances than ML-based methods for very low sequence identity thresholds, in all ontologies, improving substantially only with an increase in maximum sequence similarity. The results of information theoretic assessments at varying identity thresholds (Additional file 1: Figs. A5, A6) further highlight the promising potential of GO-LTR in annotation novel sequences with highly specific and rarely observed functional terms in the ontology.

## Discussion

In this work, we studied how different protein features can be integrated for the protein function annotation task in a multi-view learning framework. Specifically, we introduced GO-LTR, a latent tensor reconstruction model that learns the multi-way interactions between multiple protein features. Extensive evaluation across several performance measures demonstrate the competitive predictive accuracy of GO-LTR in annotating proteins.

The experimental findings show that performance improvements are seen when using feature combinations in all 3 ontologies. Notably, the incorporation of all 3 features as input resulted in the best performance in the BPO category. Modest performance degradation, however, was seen when utilizing all 3 features in the molecular function and cellular component sub-ontologies. Additionally, the integration of information derived from the motifs and families in the InterPro feature and the sequence embeddings (UniProt) resulted in a marked improvement over the use of each feature separately. These ontology-specific performance discrepancies can be attributed to the inherent intricacies of each sub-ontology.

Interestingly, even though the network data (PPI) for some proteins in our dataset were absent in the StringDB data, using the dense representation equivalent of this feature independently in CCO outperformed the case where InterPro fingerprints were used. This is likely explained by the close proximity of proteins working in concert in a living cell. Also, motifs and domain information in the InterPro fingerprints were highly predictive of functions in BPO. We posit that InterPro fingerprints are highly influential in describing larger cellular processes like those in BPO. Furthermore, the best performing linear GO-LTR model, making use of only one feature, outperformed the frequency and similarity-based baseline methods in all three sub-ontologies, thereby highlighting the practical potential of GO-LTR in annotating proteins.

Indeed, the result of further ablation studies illustrate that feature combinations are necessary in improving the generalization performance and stability of all machine learning models considered in this study. Surprisingly, while DeepGOMLP leverages residual connections between the outputs of successive layers to maintain the flow of gradients, its annotation accuracy in the 3-view experiments was not significantly better than that of GO-LTR which has no skip connections in its architecture. Additionally, the number of model parameters in the biggest GO-LTR model, the 3-view model, is several orders of magnitude less than the 1-view DeepGO counterparts.

The observations from the performance comparison based on depth and frequency of functional terms, indicate the competitive annotation accuracy of GO-LTR in predicting highly specific and rarely observed terms in the gene ontology. As the prediction of highly informative terms is desired in this task, we propose that future studies should include information content of terms with respect to the ontology, in the optimization objective.

We investigated the predictive accuracies of all competing methods on an unseen dataset. The findings show GO-LTR’s strong performance in the MFO and CCO categories of the ontology. GO-LTR, however, fell to third place in the BPO category, outperformed by NetGO3.0 and DeepGOMLP by 4.7% and 2.3% respectively. Consistent with previous works in the function prediction space, all models exhibited substantially worse performances in the BPO category compared to the results in MFO and CCO subontologies. This substantial drop in performance could be attributed to the deep and dense nature of the BPO graph, as well as the low annotation quality and high-level abstraction of its terms. Additional studies investigating this low performance phenomenon from several modeling and experimental perspectives are required.

We studied the generalizability of models to sequences at varying homologies to observed sequences in the training set. The results assert GO-LTR’s practical potential in annotating proteins that fall in the most difficult, midnight zone (< 20%) of sequence identity. This is essential because in this zone, inference via homology-based methods, which capitalize on evolutionary relatedness, provide highly unreliable and statistically uncertain results [9, 31]. Similarly, GO-LTR, like all other machine learning models outperformed homology-based BLAST and term-frequency-dependent Naive model, in the twilight (20–35%) and safe (> 40%) zones of sequence similarity. These results contextualize the importance of data-dependent models and multiple informative features in reliably predicting functional terms.

## Conclusion

In this study, we introduced GO-LTR, an automatic protein function prediction method that leverages the underlying relationships between diverse protein features in a multi-view learning framework. It relies on an efficient tensor-based estimation of model parameters. Extensive experimental validation demonstrate its high prospects in annotating proteins under several challenging conditions: generalizing to low sequence homology, rarely observed functional terms and highly specialized terms in the gene ontology.

### Supplementary information

**Additional file 1.** Contains further performance comparisons on Dataset-1 and Dataset-2, model parameters and the detailed description of the architecture of the protein language model used.

## Data Availability

The dataset used in the experiments and their descriptions are available at https://github.com/aalto-ics-kepaco/GO-LTR-prediction.

## Code availability

The source code for running the model is available at https://github.com/aalto-ics-kepaco/GO-LTR-prediction under MIT License.
