# Supplementary material for: Protein function prediction through multi-view multi-label latent tensor reconstruction
Source: BMC Bioinformatics. 2024 May 2;25:174. doi: 10.1186/s12859-024-05789-4 (PMC11067221; doi:10.1186/s12859-024-05789-4)
Supplement: Supplementary file 1 — Additional file 1. Contains further performance comparisons on Dataset-1 and Dataset-2, model parameters and the detailed description of the architecture of the protein language model used. [file 12859_2024_5789_MOESM1_ESM.pdf]

Appendix A Protein Function Prediction Through Multi-view Multi-label Latent Tensor Reconstruction.

(by Robert Ebo Armah-Sekum, Sandor Szedmak, Juho Rousu),
Department of Computer Science, Aalto University, Finland

A.1 Performance comparison on Dataset-2 (i.e. independent test set)

Dataset-2 is described in the Section 2.3 of the main text. It comprises sequences in the UniProtKB database that gained functional annotations in the period: 14.03.2023 - 24.01.2024.

Table A1 Performance evaluation on independent test set for Molecular Function Ontology. Best performing model is highlighted in bold font and second place model is underlined.

**Model**	$F_{max}(\uparrow)$	$WF_{max}(\uparrow)$	$S_{min}(\downarrow)$	**AUPRC**(↑)	**AUROC**(↑)
Naive	0.375	0.147	10.153	0.131	0.851
BLAST-partial	0.503	0.461	13.507	0.444	0.886
BLAST-full	0.615	0.524	9.305	-	0.791
DeepGOCNN	0.559	0.417	8.085	0.396	0.906
DeepGOMLP	**0.673**	**0.597**	6.529	0.601	**0.971**
NetGO3.0	0.646	0.576	6.667	0.618	0.950
GO-LTR	**0.673**	0.585	**6.179**	**0.689**	0.968

Table A2 Performance evaluation on independent test set for Cellular Component Ontology. Best performing model is highlighted in bold font and second place model is underlined.

**Model**	$F_{max}(\uparrow)$	$WF_{max}(\uparrow)$	$S_{min}(\downarrow)$	**AUPRC**(↑)	**AUROC**(↑)
Naive	0.570	0.439	7.461	0.327	0.929
BLAST-partial	0.553	0.481	14.626	0.522	0.898
BLAST-full	0.686	0.562	9.219	-	0.842
DeepGOCNN	0.716	0.584	5.927	0.301	0.939
DeepGOMLP	0.738	0.622	5.816	0.681	**0.975**
NetGO3.0	0.728	0.611	6.300	0.781	0.958
GO-LTR	**0.765**	**0.663**	**5.142**	**0.817**	**0.975**

Table A3 Performance evaluation on independent test set for Biological Process Ontology. Best performing model is highlighted in bold font and second place model is underlined.

**Model**	$F_{max}(\uparrow)$	$WF_{max}(\uparrow)$	$S_{min}(\downarrow)$	**AUPRC**(↑)	**AUROC**(↑)
Naive	0.313	0.218	27.437	0.181	0.872
BLAST-partial	0.381	0.340	87.963	0.317	0.844
BLAST-full	0.454	0.387	32.526	-	0.730
DeepGOCNN	0.412	0.309	26.846	0.331	0.898
DeepGOMLP	0.503	0.423	22.965	0.463	**0.945**
NetGO3.0	**0.527**	**0.454**	**21.916**	**0.484**	0.823
GO-LTR	0.480	0.386	22.696	0.455	0.942

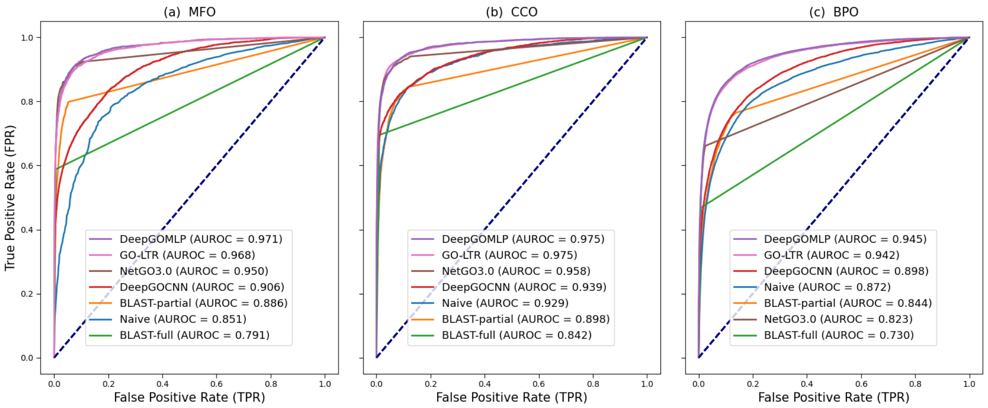

Figure A1 Performance comparison on Dataset-2 using receiver operating characteristics (ROC) curves in (a) molecular function, (b) cellular component and (c) biological process ontology. The area under the ROC (AUROC) curve is given in the legends.

A.1.1 Performance evaluation of feature combinations in GO-LTR on independent test set

Here, the model is retrained on the full training set (sequences annotated on or before 13.03.2023) and evaluated on the independent test set (sequences annotated between 14.03.2023 and 24.01.2024)

Table A4 Ablation experiment on independent test set: Effect of feature combinations to GO-LTR performance as measured by F_{max}, $\uparrow$ higher the better, in all 3 ontologies. Best performing feature combinations are highlighted in bold font.

		$\boldsymbol{F_{max}}(\uparrow)$		
**Views**	**Feature combinations**	**MFO**	**CCO**	**BPO**
1-view	InterPro	0.598	0.685	0.397
	PPI	0.418	0.596	0.334
	UniProt	0.615	0.748	0.453
2-view	InterPro + PPI	0.641	0.693	0.427
	InterPro + UniProt	**0.673**	0.764	0.476
	PPI + UniProt	0.654	**0.765**	0.473
3-view	InterPro + PPI + UniProt	0.663	0.757	**0.480**

A.1.2 Feature contributions in GO-LTR extracted from the trained model

The effect of each feature/view d is given by $\mathbf{D}^{(d)}_{\lambda_U}$ in Table 1f. The weights corresponding to the contribution of each feature in the prediction of the output is then extracted from the retrained model described in Section A.1.1. The top-100 components are then sorted in descending order of magnitude ($\lambda_1 \geq \lambda_2 \geq \cdots \geq \lambda_{100}$) and their magnitude plotted on a logarithmic scale. The components are L2-normalized.

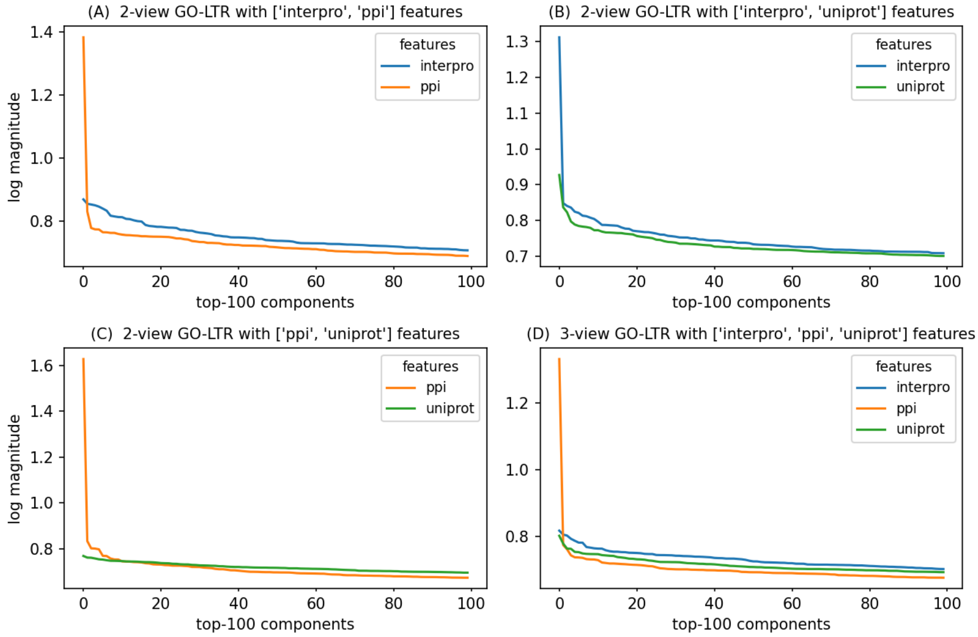

Figure A2 Feature contributions in 2-view and 3-view GO-LTR models of the Molecular Function ontology based on Dataset-2: The magnitudes of the top-100 components are plotted on a logarithmic scale.

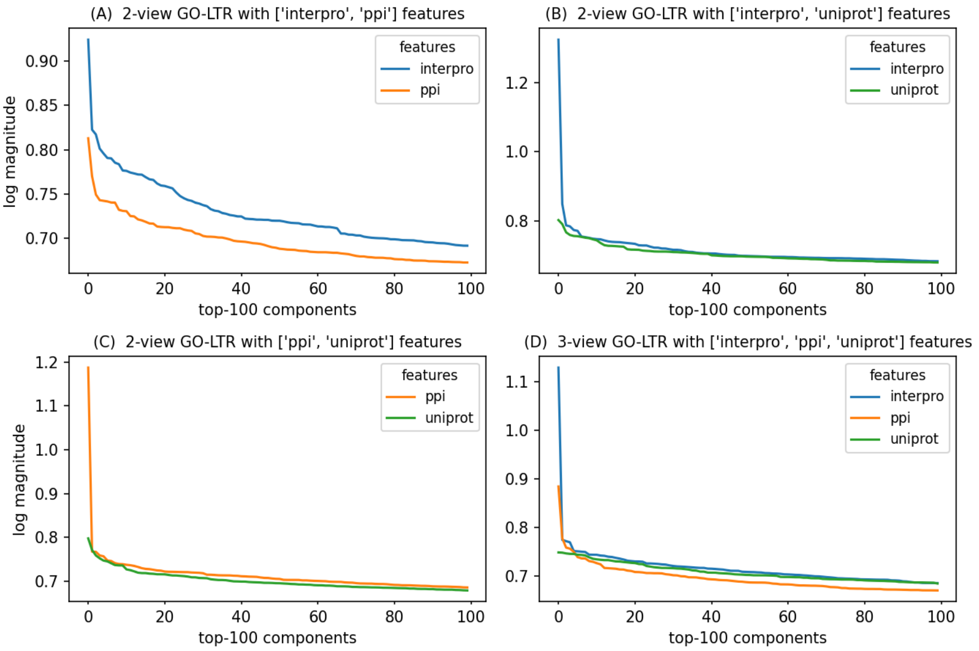

Figure A3 Feature contributions in 2-view and 3-view GO-LTR models of the Cellular Component ontology based on Dataset-2: The magnitudes of the top-100 components are plotted on a logarithmic scale.

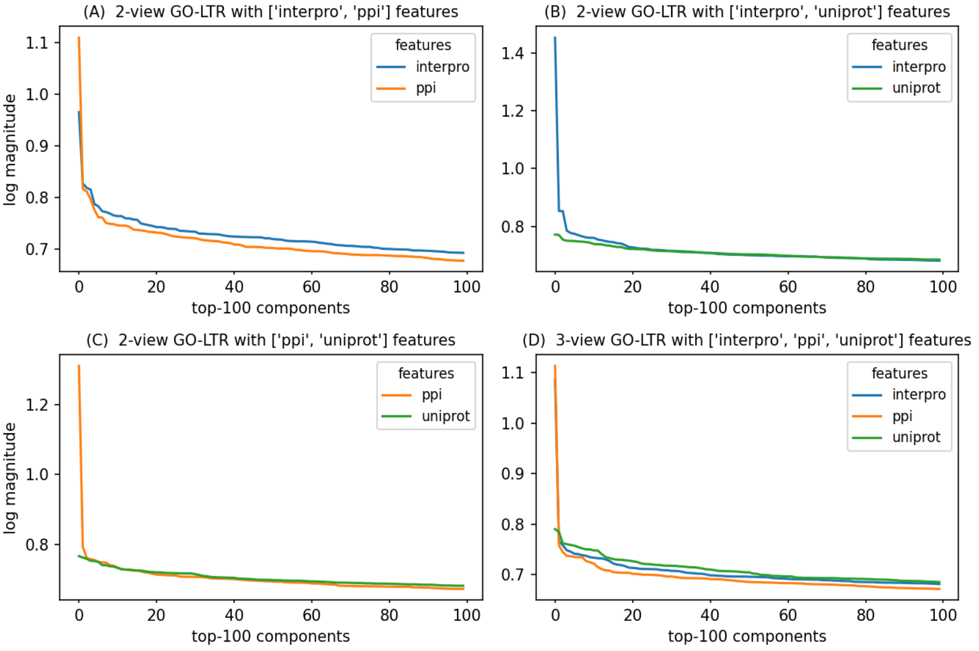

Figure A4 Feature contributions in 2-view and 3-view GO-LTR models of the Biological Process ontology based on Dataset-2: The magnitudes of the top-100 components are plotted on a logarithmic scale.

A.1.3 Performance evaluation on Dataset-2

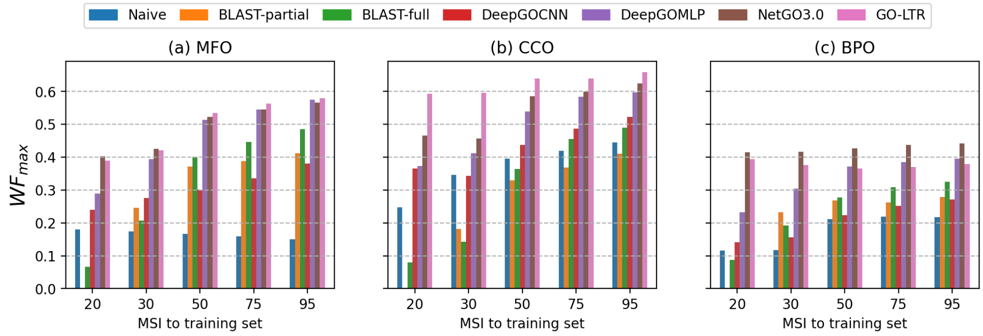

Figure A5 Performance comparison on Dataset-2 using $WF_{max}(\uparrow)$ based on groupings of sequences in the test set by their maximum percentage sequence identity (MSI) to sequences in the training set, for all ontologies. The absence of BLAST-partial in the 20% MSI cutoff is due to the absence of relevant hits among training sequences detected at an e-value of 0.001.

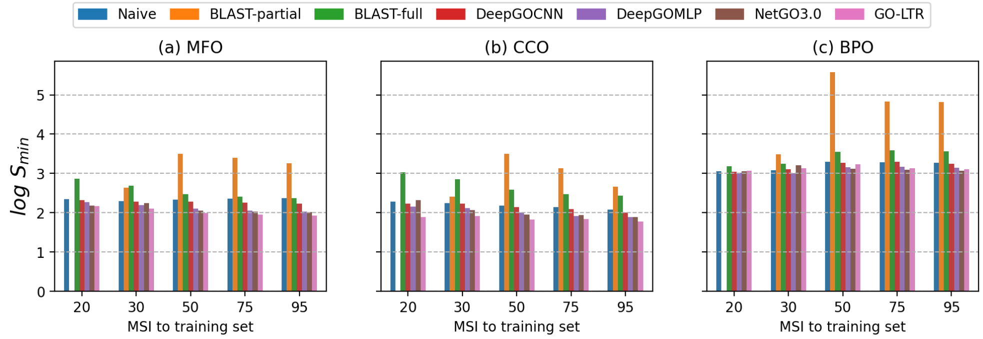

Figure A6 Performance comparison on Dataset-2 using $S_{min}(\downarrow)$ based on groupings of sequences in the test set by their maximum percentage sequence identity (MSI) to sequences in the training set, for all ontologies. The absence of BLAST-partial in the 20% MSI cutoff is due to the absence of relevant hits among training sequences detected at an e-value of 0.001.

A.2 Performance comparison on Dataset-1

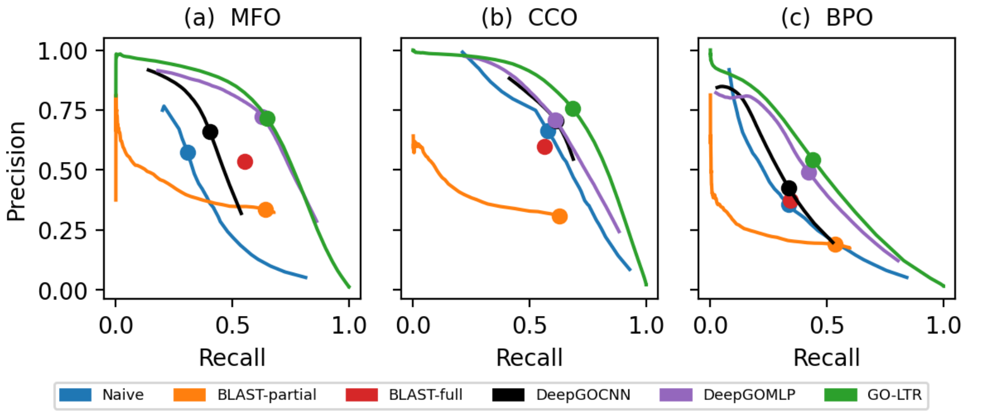

Figure A7 Mean precision-recall curves over the 10 outer cross validation splits of Dataset-1 in all ontologies. The dot on each curve indicate the precision-recall point at which the mean F_{max} was achieved.

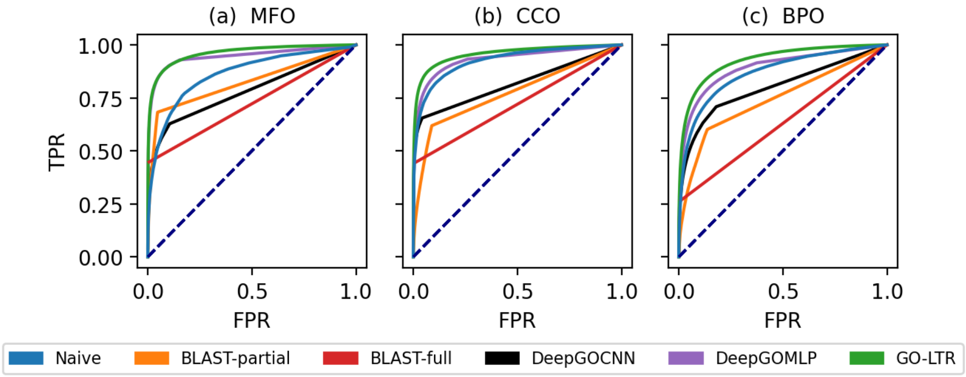

Figure A8 Mean receiver operating characteristics (ROC) curves over the 10 outer cross validation splits of Dataset-1 in all ontologies. In the figure, the mean true positive rate (TPR) versus the mean false positive rate (FPR) is plotted.

A.3 Performance comparison of 3-view Machine Learning (ML) models on Dataset-1 10-fold CV

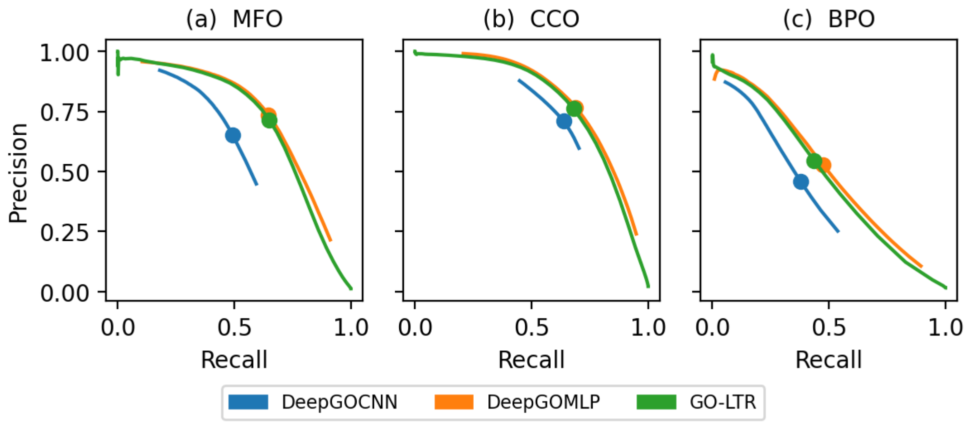

Figure A9 Mean precision-recall curves over the 10 outer cross validation splits of Dataset-1 for 3-view ML models in all ontologies. The dot on each curve indicate the precision-recall point at which the average F_{max} was achieved.

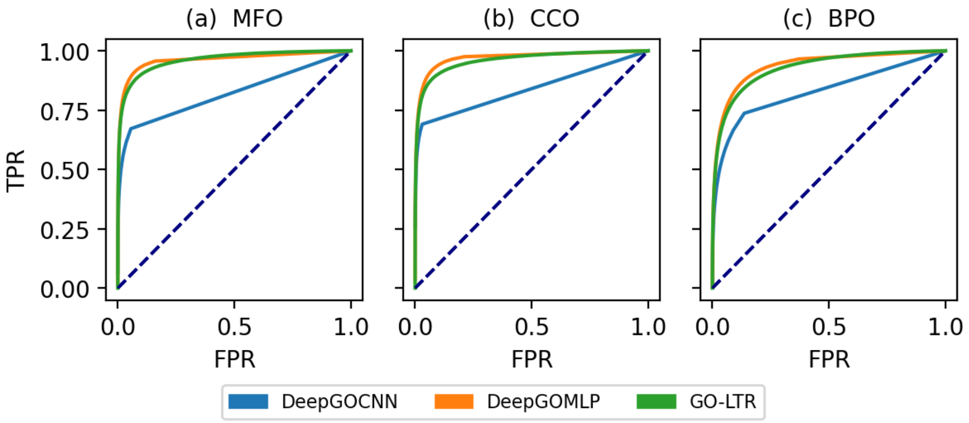

Figure A10 Mean receiver operating characteristics (ROC) curves over the 10 outer cross validation splits of Dataset-1 for 3-view ML models in all ontologies. In the figure, the mean true positive rate (TPR) versus the mean false positive rate (FPR) is plotted.

A.4 Comparison of model parameters in ML models

Table A5 Number of trainable parameters in DeepGOCNN, DeepGOMLP and 3-view GO-LTR model. Models with the least number of parameters are indicated in bold font.

Model	MFO	CCO	BPO
DeepGOCNN	20,891,400	20,726,375	23,221,225
DeepGOMLP	17,192,712	17,975,911	20,756,457
3-view GO-LTR	**3,614,400**	**3,614,400**	**3,614,400**

A.5 Architecture of ProtT5 protein language model

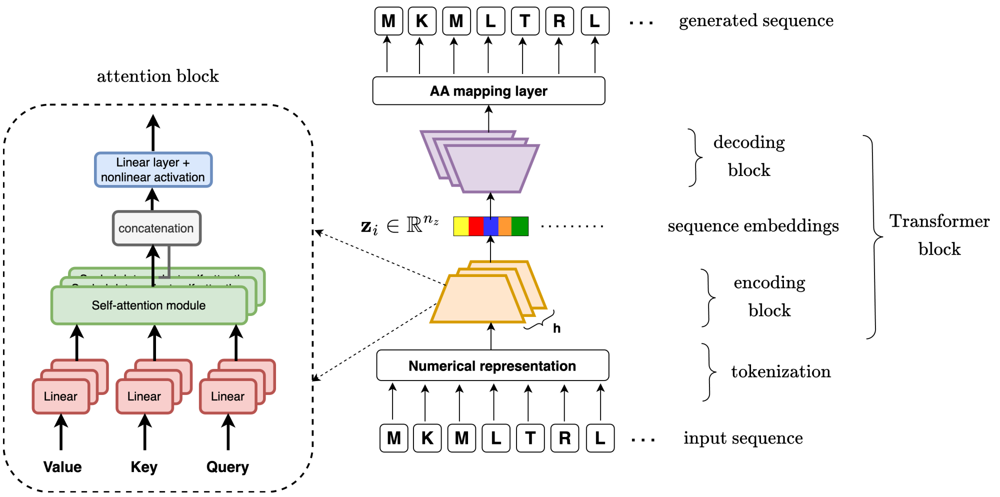

Figure A11 **Encoding block**: In the architecture, the input AA sequence is converted to a numerical representation (tokenization) for subsequent layers. The encoding block consists of several encoding units stacked on top of one another, with each learning complex relationships from the input sequence. Each encoding unit is made up of a self-attention layer and a feed-forward network (FFN). A Layer Normalization operation is computed atop the output of each attention and FFN layer. The attention layer helps to capture dependencies between each AA and the remaining residues. The are multiple attentions units (i.e. attention heads) running in parallel to learn diverse relationships in the input sequence. The FFN module learns a non-linear projection of the learned relationships into a low-dimensional space (embeddings). **Decoding block**: In a similar fashion, the decoding block comprises several decoding units stacked on top of one another, with the output of one feeding into the input for the next. The decoder generates the full-length protein sequence in an auto-regressive manner: using masked self-attention on the right-shifted output sequence, cross attention on the representations from the encoding block to generate a residue at a time by paying attention to only the residues observed up to the current time point. Just like that observed in the encoding block, there are multiple attention heads running in parallel to learn richer and diverse representations encoded by the previous layer during the sequence generation process. Finally, the numerical representations of the generated residues are then mapped back into the AA character space. We would like to note that the embeddings utilized in our studies are extracted from the encoder's output.
